# Gross and scanning electron microscopic features of the oral cavity (palate, tongue, and sublingual floor) of the Egyptian long-eared hedgehog (*Hemiechinus auratus aegyptius*)

**DOI:** 10.1186/s12917-024-04261-y

**Published:** 2024-09-28

**Authors:** Mohamed M. A. Abumandour, Basma G. Hanafy

**Affiliations:** Department of Anatomy and Embryology, Faculty of Veterinary Medicine, Alexandria University, Abees 10th Post Box: 22785, Alexandria, 21944 Egypt

**Keywords:** Egyptian long-eared hedgehog, Gemmal papillae, Hard palate, Lingual papillae, Soft palate, Scanning electron microscope

## Abstract

The study was focused on the anatomical characteristics of the Egyptian long-eared hedgehog's oral cavity by using gross and scanning electron microscopic examinations. The upper lip had an elongated T-shaped snout-like structure. The hard palate had a triangular rostral part (which had a semicircular area and a caudal ridged area with the first 3 or 4 ridges) and a caudal part (which contained seven or eight slightly oblique ridges with raphae). The diamond-incisive papilla is flanked on both sides by a groove and a fissure. The hard palate surface had glandular openings and a microplicae system. The uneven, soft palate's surface had multiple grooves and folds with 12–16 Gemmal papillae. The Gemmal papillary surface had three to four taste pores with microplicae and glandular openings. The dorsal lingual surface had six filiform subtypes: pointed (on the tip, rostral border, and median apical region), triangular (on the lateral apical and circumvallate regions), bifurcated (on the median tip only), leaf-like (on the median body region), branched (on the lateral root region), and small pointed papillae (on the median root). There were two fungiform subtypes: ovals (on the rostral border and lateral region) and rounds (on the median apical region and body). The caudal root part had a triangular arrangement of three circumvallate papillae. In conclusion, the finding confirmed its oral cavity adaptation with its insectivorous feeding habits and Egyptian environment.

**Graphical Abstract:**

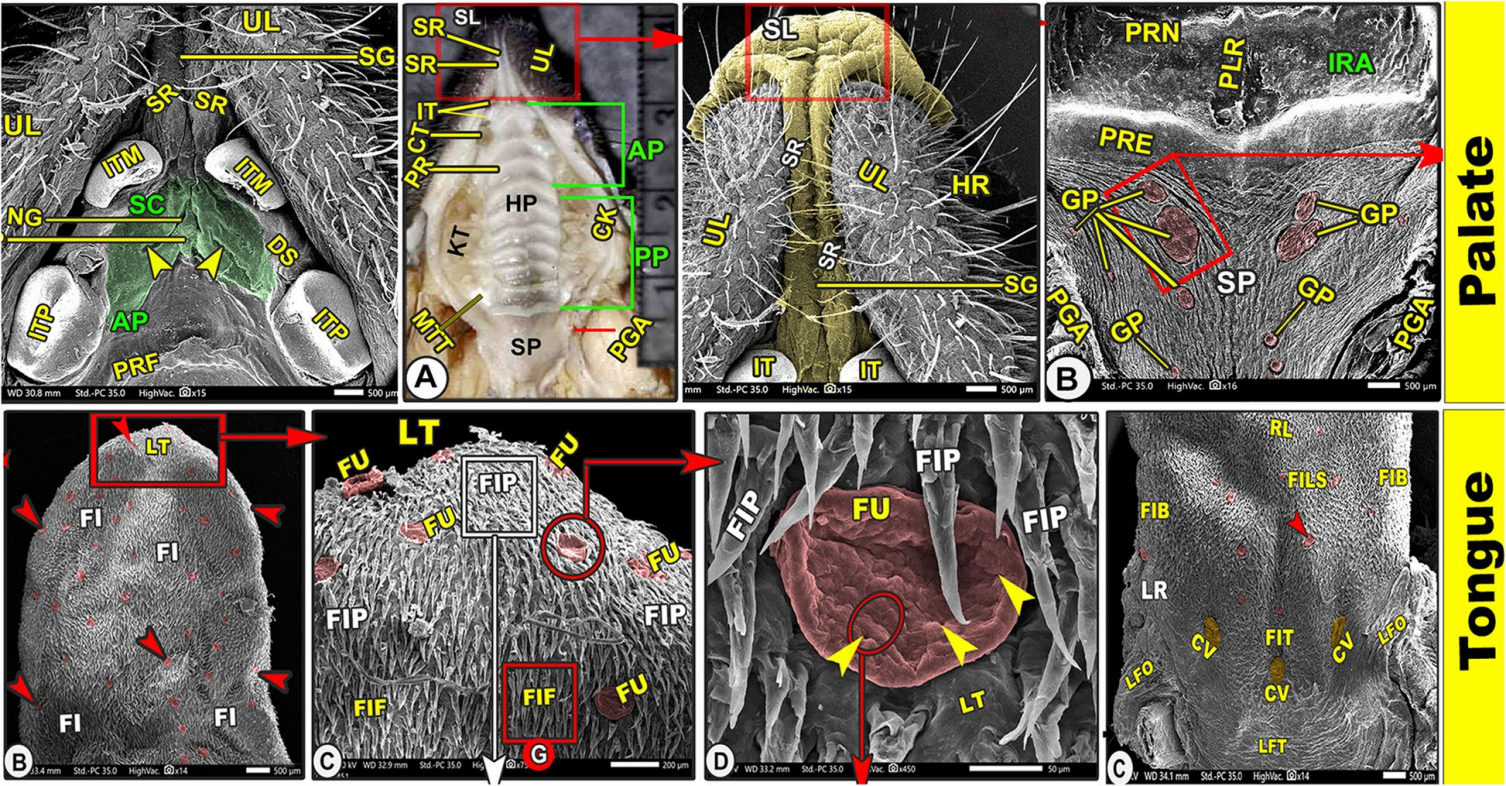

## Highlight

The hard palate had a triangular rostral part (which was divided into a semicircular area and a caudal ridged area) and a caudal part (which contained seven or eight slightly oblique ridges). The diamond-incisive papilla is flanked on both sides by a groove and a fissure. The hard palate had glandular openings and a microplicae system. The uneven, soft palate's surface had multiple grooves and folds with 12–16 Gemmal papillae. The Gemmal papillary surface had three to four taste pores with microplicae and glandular openings. The dorsal lingual surface had six filiform subtypes: pointed, triangular, bifurcated, leaf-like, branched, and small pointed papillae. There are two fungiform subtypes: ovals (on the rostral border and lateral region) and rounds (on the median apical region and body). The caudal root part had a triangular arrangement of three circumvallate papillae.

## Introduction

Based on their eating habits, we can classify mammals as frugivorous, carnivorous, herbivorous, insectivorous, or omnivorous [1–3]. Hedgehogs are small insectivores with a spiny coat that are occasionally kept as pets and sometimes used therapeutically in certain countries. The Egyptian long-eared hedgehog, *Hemiechinus auratus aegyptius* (Geoffroy Saint-Hilaire, 1803), is one of the six species of Nile spiny hedgehogs and a member of the *Erinaceidae* family. According to Massoud and Abumandour [4], *Hemiechinus auratus* can be found throughout the Middle East (including Egypt and Libya), the Cypriot islands, and coastal semi-desert areas of Central Asia. *H. auritus* is a nocturnal terrestrial animal that loves mesic environments, including gardens, fields, and olive groves. According to Kingdon, et al. [5], it predominantly consumes insects, worms, and tiny invertebrates. Hedgehogs consume a wide range of invertebrates, including fruit, mushrooms, insects, earthworms, snails, seldom hatched birds, frogs, small reptiles, and small birds [6]. Hedgehogs are sufficiently resistant to the venom of reptiles to be able to feed on them, even if they are not totally immune to it [6].

The tetrapod's oral cavity plays a crucial role in determining the feeding strategy. However, the majority of previously published data is devoted to the description of the tongue and shows how its structure, papillae, and shape are related to feeding behaviors [4]. The palate, in contrast, is thought to be the oral cavity's underappreciated structure by the majority of published data [7]. Mammals can be classified as frugivorous, carnivorous, herbivorous, insectivorous, or omnivorous based on their eating habits [1, 3]. The ventral surface of the hard palate, forming the oral cavity floor, has an essential role in food intake and fixation during the mastication process [7]. The structural components of the hard palate are the palatine ridges, palatine raphe, and incisive papilla [8].

In various mammalian species, the highly muscular tongue exhibits distinct anatomical variations [9]. In order to carry out a variety of tasks, such as obtaining, manipulating, swallowing, and processing food, there are lingual differences in the gross and microscopic structures [10]. Numerous studies have been conducted on the tongues of primates, including rodents [11–14], porcupines [15], bats [1], hedgehogs [16], rabbits [3, 17], and primates [18, 19]. There are numerous lingual papillae that have evolved to accommodate differences in dietary requirements, environmental conditions, and food particle types on the dorsal epithelial surface of the various mammalian tongues [20]. The distribution patterns, morphologies, and densities of lingual papillae vary among mammalian species [1, 10]. According to their roles in the acquisition, manipulation, and movement of food particles towards the esophageal entrance, the mammalian papillary system is divided into mechanical (filiform and conical) and gustatory (fungiform and circumvallate) papillae [19].

The available data on the anatomical features of the Egyptian long-eared hedgehog's oral cavity (*Hemiechinus auratus aegyptius*) is limited. So, our study is prepared to give gross and scanning electron microscopic characteristics of the oral cavity, including its palate, tongue, and sublingual floor, of the Egyptian long-eared hedgehog. To support earlier studies and provide additional information on how the oral cavity adapts to various feeding schedules, new research was needed. In our work, we used the mammal *Hemiechinus auratus aegyptius* to address a number of unresolved problems with regard to modifications to the oral cavity in various Egyptian mammals. Finally, we compared our findings with previously published data on other hedgehog species and other insectivorous mammals.

## Materials and methods

### Animal’s handlings

Seven adult Egyptian long-eared hedgehogs (*Hemiechinus auratus aegyptius*) of both sexes were successfully caught alive in Egypt's Matrouh Governorate. The collected Egyptian long-eared hedgehogs were kept in the animal housing of the anatomy and embryology department, Faculty of Veterinary Medicine, Alexandria University, Egypt, following the guidelines established for the ‘Sampling protocol for the pilot collection of catch, effort, and biological data in Egypt’ [21]. Housing included a hedgehog’s cage with a temperature range of 22–24 °C and 55–70% humidity with an alternative regular light and dark cycle. The hedgehogs were provided by water, and suitable feeding materials such as crickets, mealworms, cockroaches, earthworms, superworms, hornworms, and other prey animals can all be found in diets. The animals were euthanized in a CO^2^ room, followed by cervical dislocation, before the collection of samples. This technique was carried out by a licensed zoologist in conformity with local, national, and international ethical standards and was examined for any anatomical anomalies.

### Morphological and morphometric examinations

The oral cavities were divided carefully into upper and lower parts; the samples that were being evaluated were brought to the lab's facilities on ice. The oral cavity contents (tongue with its sublingual floor and palate with its hard and soft parts) of the Egyptian long-eared hedgehog were immediately removed carefully and fixed in a 10% neutral buffered formalin saline solution. The well-fixed oral cavity contents were examined grossly and under a Wild M3 stereomicroscope (*Olympus VM VMF 2x, Eyepiece 10* × *Stereo Microscope, Japan*) to obtain more magnified images to facilitate their description. Then, these morphological oral cavity features were photographed by a digital camera (*Canon IXY 325, Japan*) after the different dimensions were recorded. The anatomical terminology was prepared in accordance with Nomina Anatomica Veterinaria [22].

### Scanning electron microscopy (SEM) description

The oral cavity contents (tongue with its sublingual floor and palate with its hard and soft parts) of the Egyptian long-eared hedgehog were prepared for the SEM according to Abumandour, et al. [23]. The examined samples were rinsed in 2.0% glutaraldehyde dissolved in 0.1 M phosphate buffer at pH 7.4. Then, they were cut and fixed in (2% formaldehyde and 1.25% glutaraldehyde in 0.1 M sodium cacodylate buffer, pH 7.2 at 4 C. Samples were washed in (0.1 M sodium cacodylate containing 5% sucrose, processed by tannic acid), then dehydrated in ascending grades of ethanol for 15 min each (50%, 70, 80, 90, 95, and 100% ethanol), then held and processed for 2 h at room temperature [7, 24]. Finally, the samples were dried in carbon dioxide, then attached to stubs with colloidal carbon and coated with gold palladium in a sputtering device. SEM analysis was carried out with a JEOL JSM 6510 lv SEM unit at the Laboratory of Electron Microscopy at the Faculty of Science, Alexandria University, Egypt.

### Digital coloring of scanning electron microscopic images

We digitally colored the scanned electron microscopic images using the Photo Filter 6.3.2 program to recognize the different structures. Several authors [18, 25, 26] have applied this method.

## Results

### Roof of oral cavity (*cavitas oris*)

#### Upper lip with its snout-like structure

**Fig. 1:**
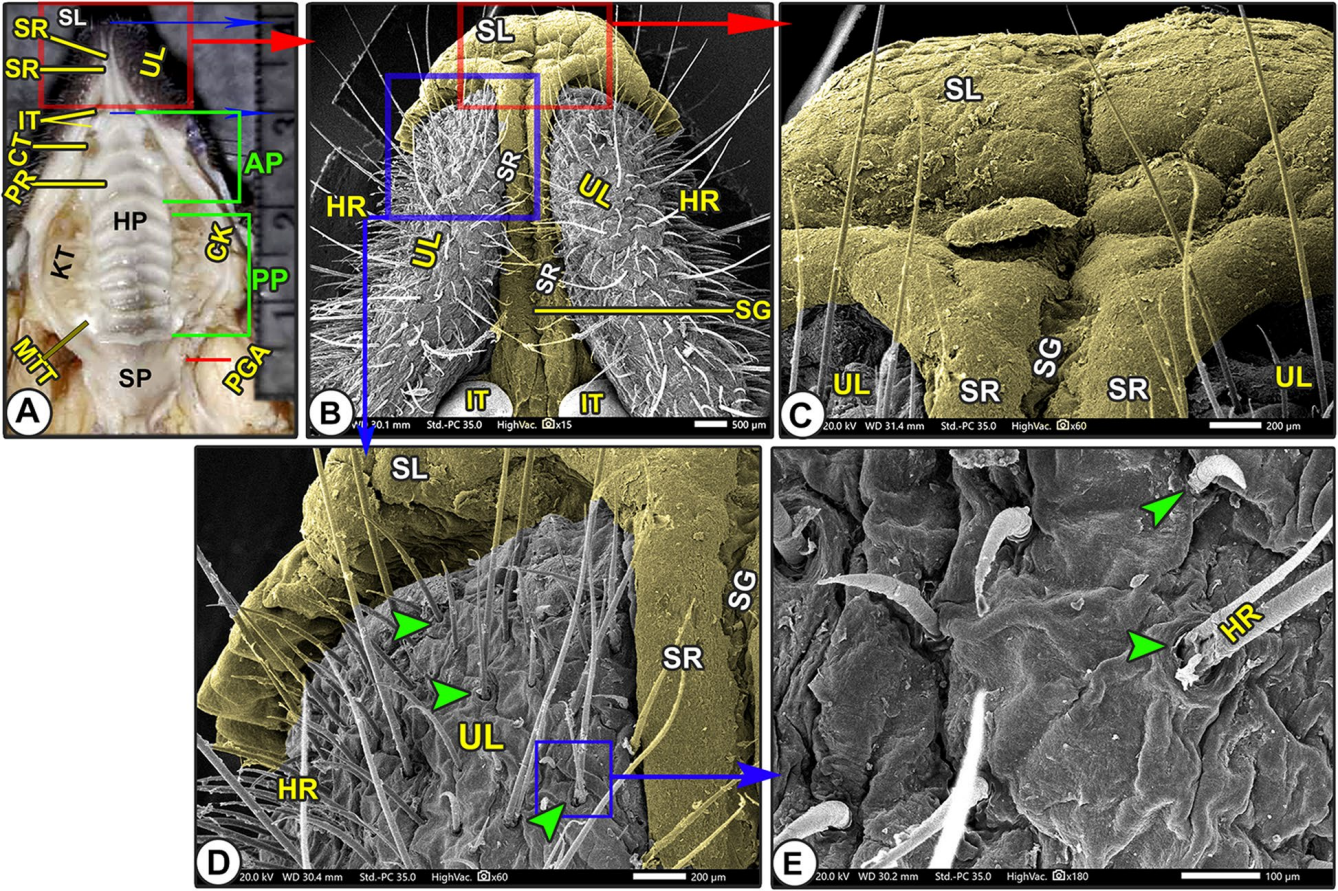
Morphological image of the oral cavity roof of the Egyptian long-eared hedgehog. (View **A**) represented the gross, and (Views **B**–**E**) represented the SEM observations to show: hard palate (HP) with its rostral (AP) and caudal (PP) parts, soft palate (SP), hair (HR) with its elevated origin (green arrowheads), check (CK), check teeth (KT), canine teeth (CT), and incisive teeth (IT). Notice the upper lip (UL) had a snout-like structure (SL) with its longitudinal ridges (SR) and groove (SG)

**Fig. 2:**
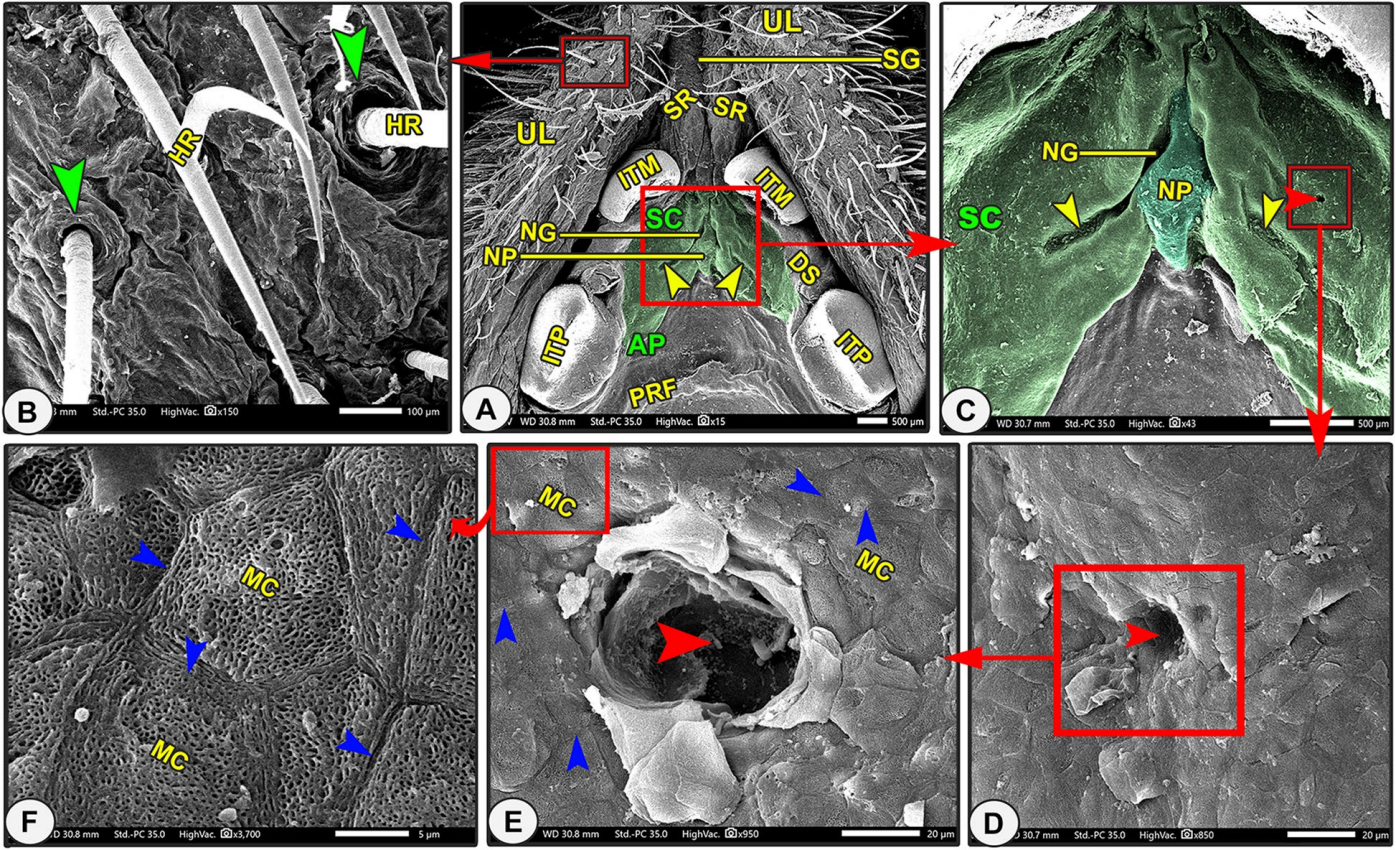
Scanning electron microscopic image of the oral cavity roof of the Egyptian long-eared hedgehog. The rostral palatine part (AP), semicircular area (SC), incisive papillae (NP) surrounded by groove (NG) and oblique fissure (yellow arrowheads), first palatine groove (PRF), and hair (HR) with elevated origin (green arrowheads) are all depicted**.** A diastema is an interdental space (DS) between the median (ITM) and peripheral (ITP) incisive teeth. Notice that the upper lip (UL) carried a snout-like structure (SL) with its longitudinal ridges (SR) and groove (SG). The opening of the palatine gland (red arrowheads) is surrounded by microplicae cells (MC) with their border (blue arrowheads)

Grossly, the elongated upper lip (*Labium superius oris*) was a characteristic feature of the Egyptian long-eared hedgehog's oral cavity due to the presence of the elongated T-shaped snout-like structure that divided the upper lip into right and left elongated parts (Fig. 1A/UL, SL). SEM observations revealed that the T-shaped snout-like structure was formed from a rostrally convex cap-like structure that was connected with two longitudinal columns in between narrow grooves that separated the upper lip into parts: right and left (Fig. 1B-D/SL, SR, SG). With high magnification, the cap-like structure had a slightly lobulated appearance with a small number of scales, while the two longitudinal columns had a smooth surface (Fig. 1C-D/SL, SR, SG). Moreover, the two lateral labial parts (right and left) were characterized by a corrugated surface with numerous sensory and ordinary hairs of different sizes that emerged from a crescent-shaped top (Figs. 1, 2B/HR, green arrowheads).

## Palate *(Palatum)*

### Hard palate* (Palatum durum)*

#### Gross anatomy

**Fig. 3:**
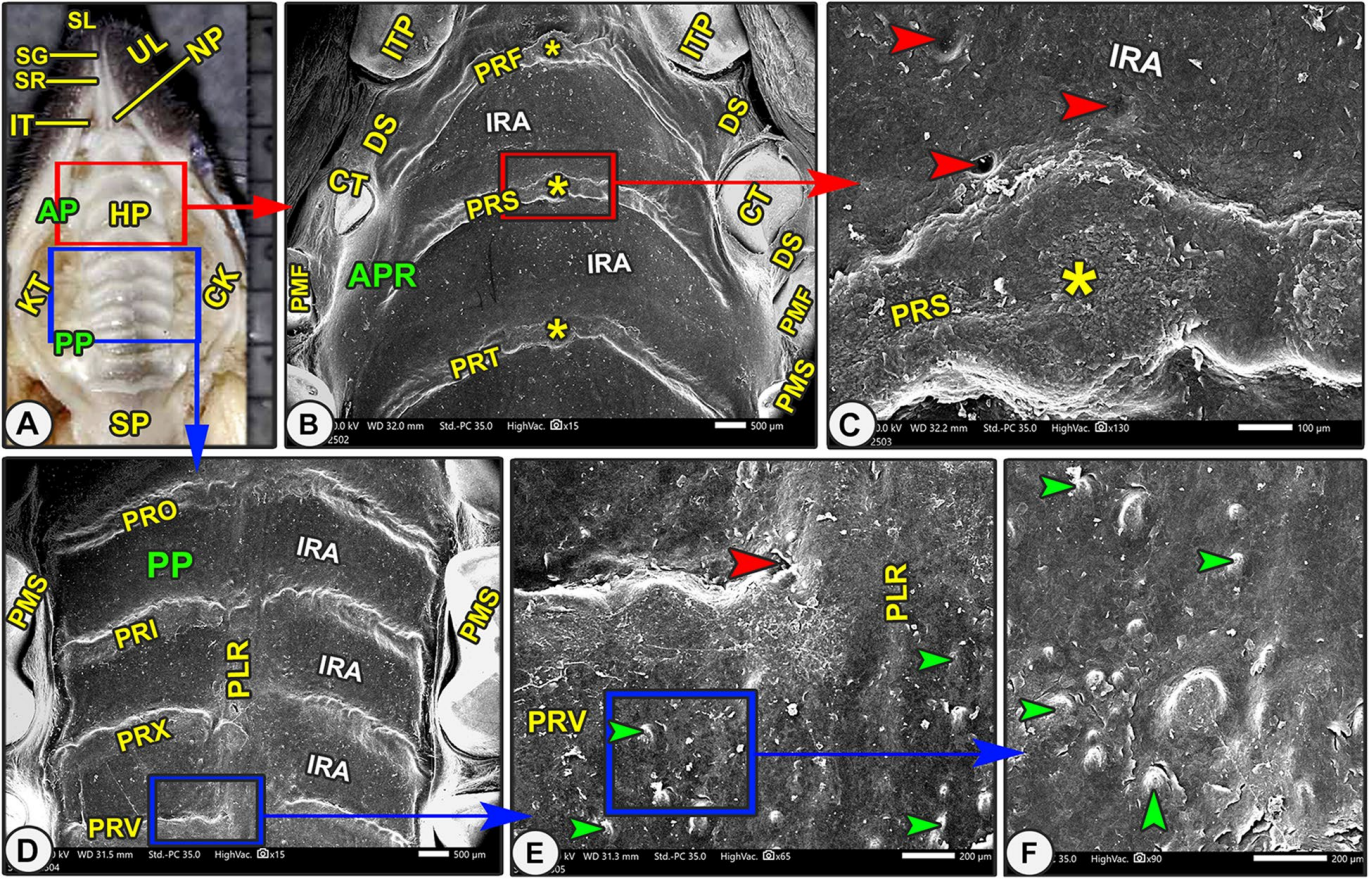
Scanning electron microscopic image of the oral cavity roof of the Egyptian long-eared hedgehog. The hard palate (HP) with its rostral (AP) and caudal (PP) parts, the soft palate (SP), check (CK), check teeth (KT), and incisive teeth (IT). The 1st (PRF), 2nd (PRS), 3rd (PRT), 4th (PRO), 5th (PRI), 6th (PRX), and 7th (PRV) palatine ridge (PRT), median tubercle (stars), and palatine raphe (PLR) have inter-ridged spaces (IRA). A diastema is an interdental space (DS) between the peripheral incisive teeth (ITP) and canine teeth (CT), 1st (PMF), and 2.^nd^ (PMS) premolar teeth. There were some openings of the palatine gland (red arrowheads) and palatine tubercles (green arrowheads)

**Fig. 4:**
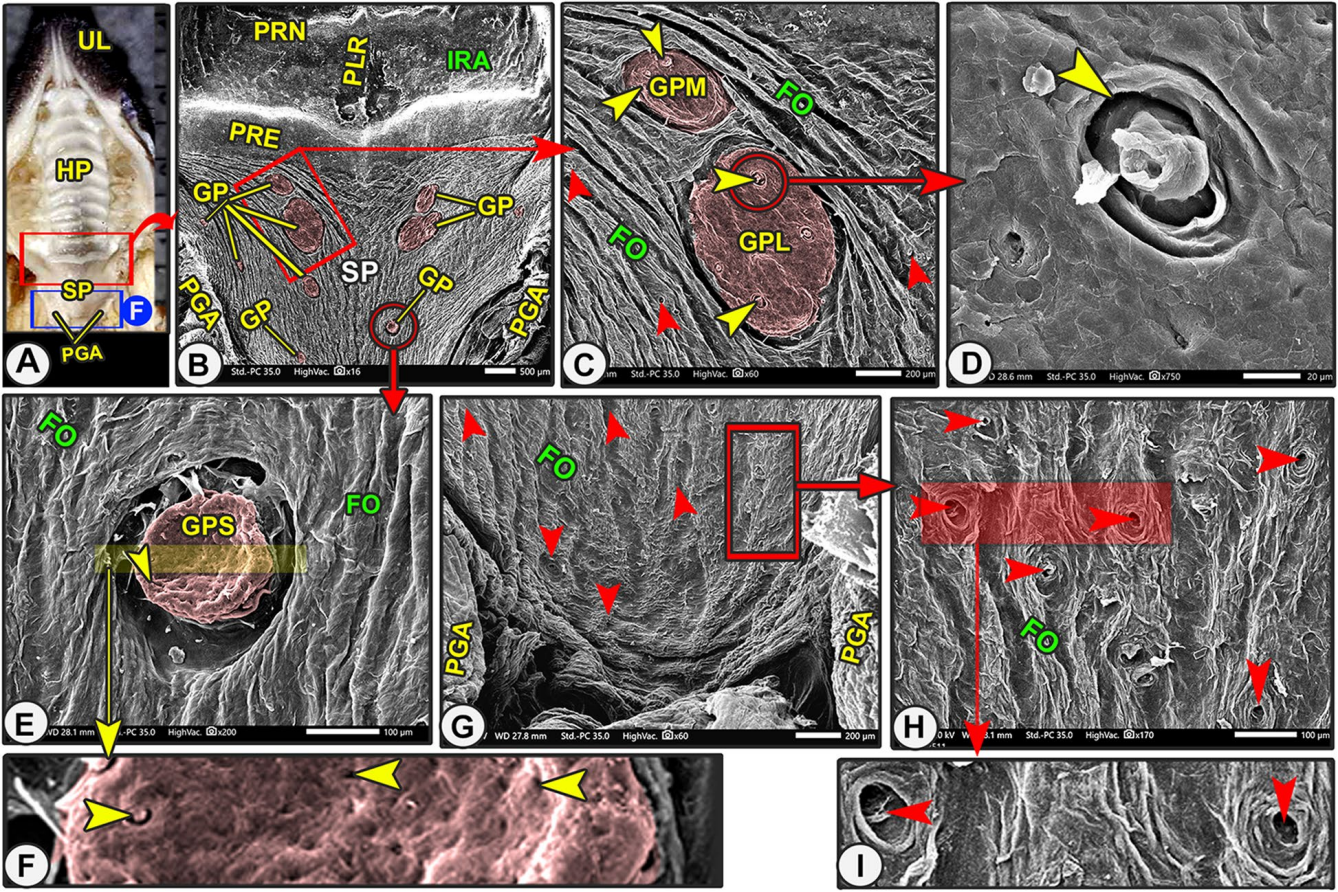
Gross (View **A**) and Scanning electron microscopic (Views **B–I**) image of the oral cavity roof of the Egyptian long-eared hedgehog. The upper lip (UL), hard palate (HP) with its 9th and 10.^th^ palatine ridges, soft palate (SP) with its palatoglossal arch (PGA), palatine gland opening (red arrowheads), Gemmal papillae (GP) that were classified into, large oval (GPL), moderate oval (GPM), and small rounded (GPS) papillae and carried taste pores (yellow arrowheads)

The elongated hard palate was begun behind the incisor teeth and from the caudal termination of the two longitudinal columns of the snout-like structure rostrally to the third molar teeth caudally (Figs. 1A, 3A, 4A/HP, SR, and MTT). The hard palate had two surfaces: the nasal (dorsal surface) and the oral (ventral) with its ridges (Figs. 1A, 3A, 4A/HP, PT). The hard palate was divided according to its shape and the curvature of the transverse palatine ridges into two parts: the triangular small rostral part of (0.9 ± 0.012 mm) in length and the straight large quadrilateral caudal part of (1.6 ± 0.23 mm) in length (Figs. 1A, 3A, 4A/HP, AP, PP). The rostral part formed 36% and the caudal part formed 64% of the total length of the hard palate.

#### Scanning *electron* microscopic anatomy

The rostral palatine part was lengthened by the space named the "diastema" that existed between the incisors and molariform teeth, in addition to the presence of the T-shaped snout-like structure of the upper lip (Figs. 2A, 3B/AP, DS, SL). The triangular rostral part was extended from the incisor teeth and the caudal termination of the two longitudinal columns of the snout-like structure rostrally to the rostral border of the second premolar teeth (Figs. 2A, 3B-C/AP, ITM, SR, PMS). The triangular rostral part was subdivided into a rostral slightly elevated semicircular area (before the beginning of the appearance of the transverse palatine ridges) and a caudal ridged area (Fig. 2A/AP, SC, APR). High magnification of the semilunar area revealed a small number of the palatine salivary gland openings and a system of microplicae with unique micropores and cell outlines (Fig. 2C-F/SC, red and blue arrowheads, MC). After that, the caudal ridged area had the first three or four rostrally curved transverse palatine ridges, which began to appear with no groove or crest palatine raphae separating the right and left transverse palatine ridges (*Rugae palatinae*) from each other, instead, there was a median palatine tubercle (Figs. 2A, 3B-C/APR, PRF, PRS, PRT, star).

Incisive papillae divided the semilunar area into two equal halves, right and left (Fig. 2A-C/SC, NP). The diamond-shaped incisive papilla was located at the median position of the rostral semicircular area, just parallel to the caudal border of the median first pair of incisive teeth until the beginning of the rostral border of the second pair of incisive teeth (Fig. 2A-C/SC, NP, ITM). This papilla was flanked on both sides by a palatine groove with one oblique longitudinal fissure on each side of its lateral surface (Fig. 2A-C/NP, NG, yellow arrowheads).

The straight large quadrilateral caudal part of the same width contained the nearly straight or slightly oblique seven or eight palatine ridges (Figs. 3D, 4B/PP, PRI, PRX, PRV, PRN, and PRT). The direction of the palatine ridges in this part was caudolateral, except the last one had an anteriolateral direction. The last palatine ridge was considered an anatomical landmark, illustrating the separation between the hard and soft palates (Fig. 4B/PRT, SP). The caudal palatine part was divided into equal halves by a median slightly projected crest named the palatine raphae (*Raphe palati*), separating the right and left transverse palatine ridges from each other (Figs. 3D, 4B/PP, PLR). At high SEM magnification of the hard palate, there were numerous palatine salivary gland openings in the inter-rugal space and absent from the palatine ridges (Fig. 3C, E/red arrowheads). Furthermore, there are few palatine conical papillae (Figs. 3E-F/IRA, green arrowheads).

## Soft palate (*palatum molle*)

### Gross anatomy

The soft palate was the caudal continuation of the hard palate that extended from the beginning of the rostral border of the second pair of molar teeth and the caudal edge of the last palatine ridge rostrally to about the middle of the epiglottis caudally **(**Figs. 1A, 3A, 4A**)**. Its width was somewhat reduced caudally, reaching (0.6 ± 0.0012 mm) and (0.4 ± 0.0023 mm) in width at its beginning and its ending, respectively. The right and left palatoglossal arches, which constituted the lateral borders of the pharyngeal entrance (*Aditus pharyngis*) between the oral cavity and the pharynx, connected the soft palate laterally to the lingual root (Figs. 1A, 4A/PGA). The soft palate had two surfaces: the ventral (oral) and the dorsal (nasal) surfaces.

### Scanning *electron* microscopy

The uneven, soft palate's oral surface had multiple oblique grooves and folds in the caudomedian direction; furthermore, these folds were interconnected with each other by short, small transverse ridges (Fig. 4/SP, FO). The soft palate's oral surface had 12–16 oval or round Gemmal papillae that were classified according to their size and shape into, large oval, moderate oval, and small rounded papillae. From the palatal mucosa's oral surface, the Gemmal papilla protrudes slightly (Fig. 4B-C,E/GP, GPL, GPM, GPS). The high magnification of the papillary surface revealed three to four taste pores with the microplicae system, with unique micropores and cell outlines on the irregular surface of these papillae (Fig. 4C-I/yellow arrowheads**)**. There were numerous openings of the palatine salivary glands, especially at their caudal part, in which these openings were surrounded by mucosal folds (Fig. 4C-I/red arrowheads). The different numbers and dimensions of the soft palate and the three different sizes of Gemmal papillae were recorded in Tables 1 and 2.

**Table 1:** Represent the average dimensions of the hard and soft palate (mm) of the Egyptian long-eared hedgehog (*Hemiechinus auratus aegyptius*)

	**Mean ± SD (cm)**
**The total length of the hard palate**	2.5 ± 0.02
**Length of the anterior part of the hard palate**	0.9 ± 0.012
**Length of the caudal part of the hard palate**	1.6 ± 0.23
**Length of the incisive papillae**	0.91 ± 0.074
**Width of the incisive papillae**	0.041 ± 0.011
**Width of the hard palate at the rostral part (At the interdental space between 1st and 2nd incisive teeth)**	0.5 ± 0.023
**Width of the hard palate at the middle part** (At the interdental space between 1st and 2nd incisive teeth)	1.1 ± 0.034
**Width of the hard palate at the caudal part** (At the interdental space between 1st and 2nd incisive teeth)	0.8 ± 0.021
**Number of palatine ridges**	10–12 pairs
**Length of the soft palate**	0.7 ± 0.002
**Width of the soft palate (at its beginning)**	0.6 ± 0.0012
**Width of the soft palate (at its end)**	0.4 ± 0.0023

**Table 2:** Represent the average dimensions of the different sized gemmal papillae on the oral (ventral) surface of the soft palate (um) of the Egyptian long-eared hedgehog (*Hemiechinus auratus aegyptius*)

**Gemmal papillae**	Number	Dimension	**(um)**
**Large Oval Gemmal papillae** (At the rostral half of the soft palate)	**Two**	**Major axis**	0.276 ± 0.013
		**Minor axis**	0.192 ± 0.003
**Medium Oval Gemmal papillae** (At the rostral half of the soft palate)	**Four**	**Major axis**	0.152 ± 0.004
		**Minor axis**	0.097 ± 0.001
**Medium round Gemmal papillae** (At the lateral side and the caudal half of the soft palate)	**Ten**	**Diameter**	0.045 ± 0.001

### Floor of oral cavity

#### Lower lip (*Labium inferius oris*) and cheek (*buccae*)

**Fig. 5:**
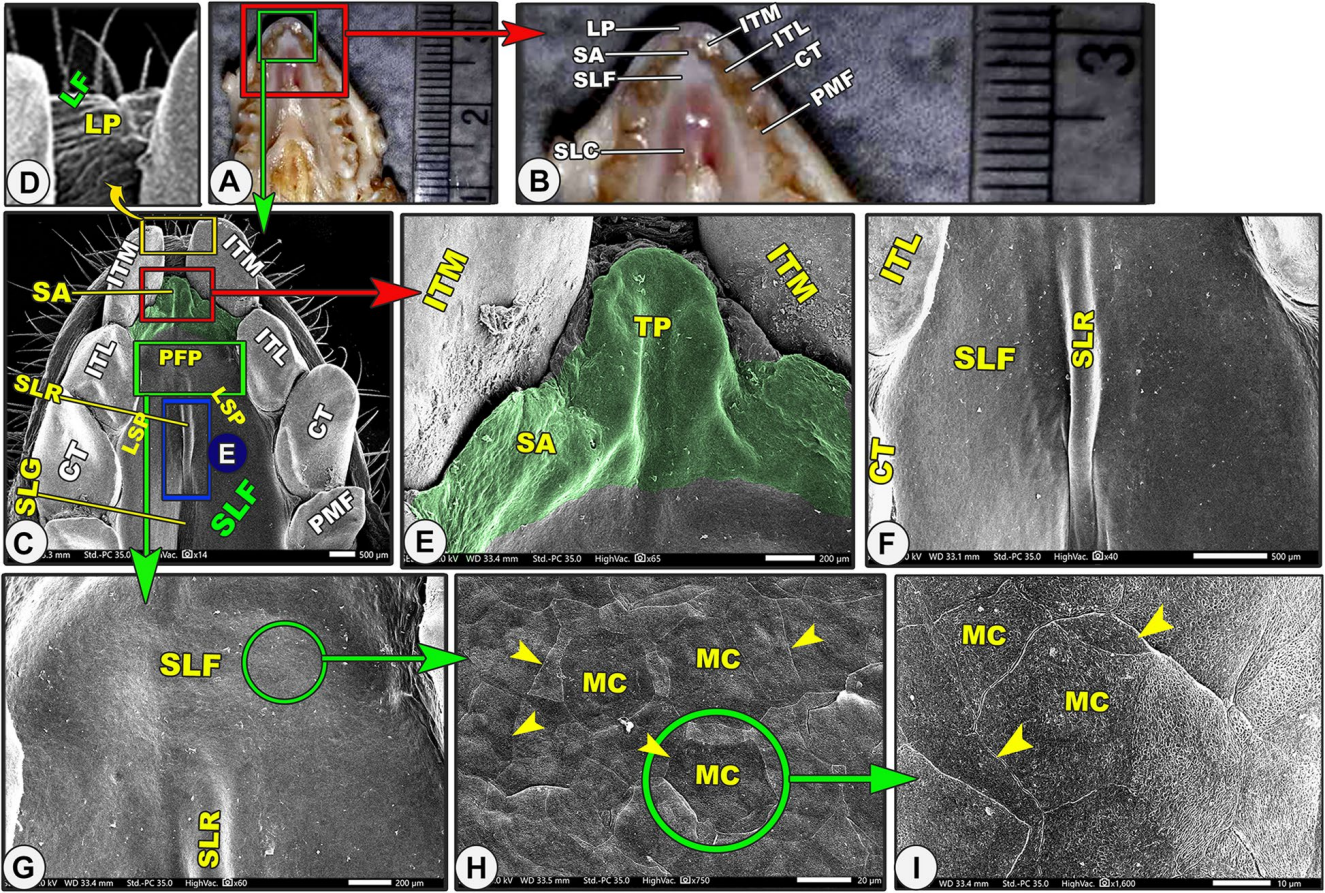
Gross (Views **A**–**B**) and scanning electron microscopic (Views **C**–**I**) images of the oral cavity floor of the Egyptian long-eared hedgehog. The lower lip (LP), median (ITM) and lateral (ITL) incisive teeth, canine teeth (CT), 1.^st^ premolar teeth (PMF), sublingual floor (SLF) with its semicircular area (SA) with its tongue-like projection (TP), prefrenular (PFP) and lateral (LSP) recess, median sublingual ridge (SLR) and groove (SLG), and sublingual caruncles (SLC). Note the microplicae cells (MC) with their cellular border (yellow arrowheads)

**Fig. 6:**
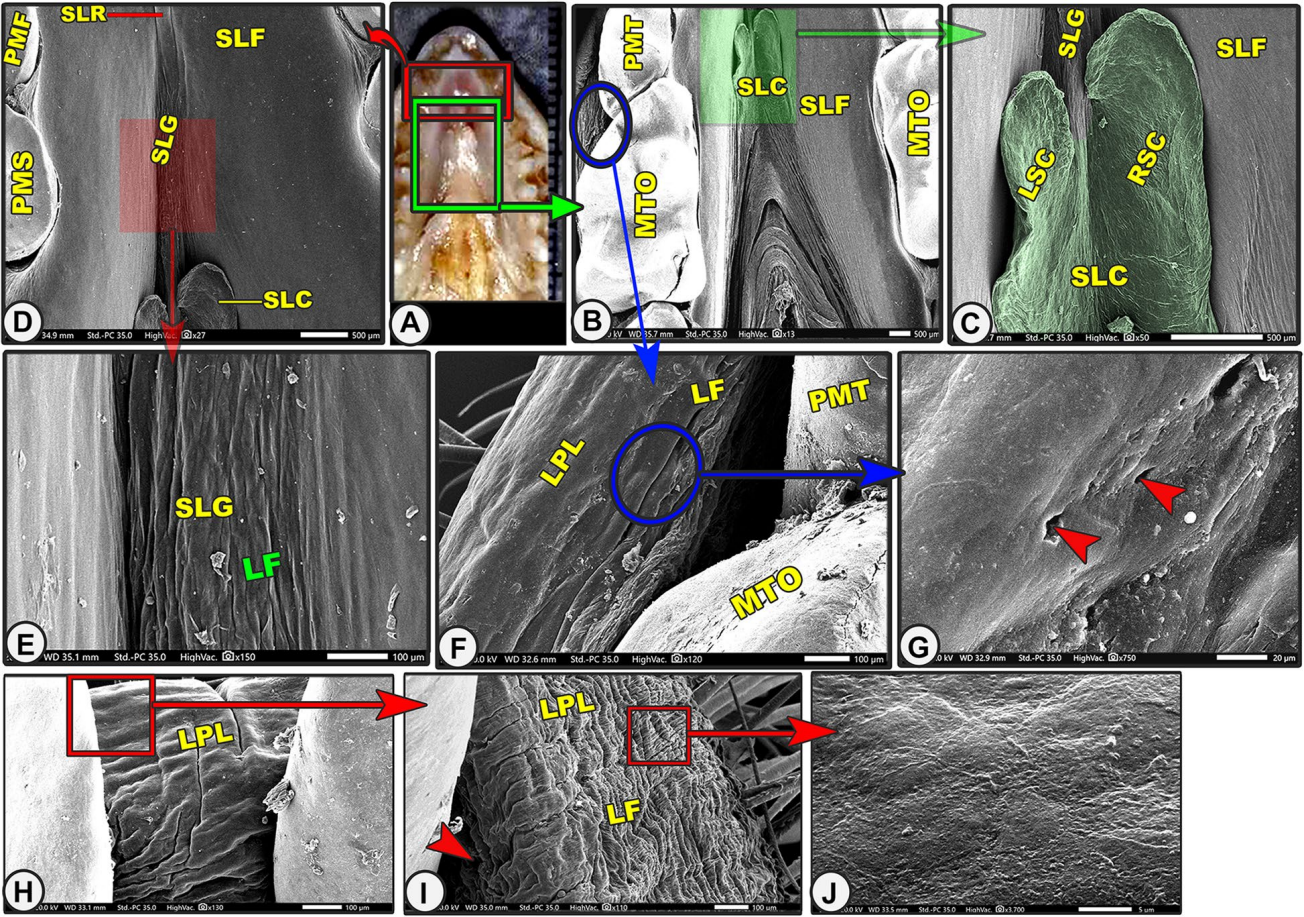
Gross (View **A**) and scanning electron microscopic (Views **B**–**J**) images of the oral cavity floor of the Egyptian long-eared hedgehog. The lateral part of the lower lip (LPL) with its labial gland openings (red arrowheads) and labial folds (LF), 1st premolar teeth (PMF), 2nd premolar teeth (PMS), 3rd premolar teeth (PMT), 4.^th^ premolar teeth (PMS), sublingual floor (SLF) with its median sublingual ridge (SLR) and groove (SLG), and sublingual caruncles (SLC) that were classified into right (RSC) and left (LSC)

Grossly, the lower lip was U-shaped in appearance and not at the same level as the upper one due to the presence of the upper T-shaped snout-like structure of the upper lip **(**Fig. 5A-B/LP). The SEM observation revealed that the lower lip and cheek carried numerous longitudinal and transverse microfolds with numerous hairs of different sizes (Fig. 5C-D/LP, LPL, LF). With high magnification, there were slightly projected cell outlines on the irregular surface (Fig. 5I). The rostral surface of the cheek had numerous openings for the buccal salivary glands (Fig. 6G-I/red arrowheads).

### Sublingual floor

It was the space located under the tongue that corresponded to the tongue shape. The sublingual floor of the oral cavity had a crescentic form and was divided into two laterals and one rostral prefrenular recess (Fig. 5C/SLF, PFP, LSP). The rostral prefrenular part was located beneath the lingual apex and was bound ventrally by the body of the mandible, laterally by the rostral part of the lower interdental space, and rostrally by the lingual surface of the incisors (Fig. 5C/PFP). The sublingual floor had a short triangular shape, tapered directly after the incisors, and was broad caudally at the level of the frenulum linguae (Fig. 5C/SLF). The rostral prefrenular recess was situated just caudal to the semilunar area. This semilunar area corresponded to the upper semilunar area of the hard palate and had a tongue-like projection on the rostral median interdental space between the two rostral incisive teeth (Fig. 5C/SLF, SA, TP).

The sublingual space was divided into two equal halves by the median projected sublingual ridge rostrally (under the lingual apex) that disappeared into the groove at the middle and the two sublingual caruncles caudally (under the lingual body) (Figs. 5C-F, 6A-D/SLF, SLR, SLG, SLC). The high magnification of the two halves of the smooth sublingual space and the median sublingual ridge revealed the presence of the microplicae system with cell outlines on the irregular surface of these papillae, while the sublingual groove had numerous microfolds (Figs. 5G-I, 6E/MC, yellow arrowheads, FL). The two sublingual caruncles were oval, with the right one (1.839 ± 0.145 um) being longer than the left one (1.495 ± 0.131 um), as shown in (Fig. 6A-D/SLC, LSC, RSC). The two sublingual caruncles were connected in the middle by a median, slightly projected ridge, but each caruncle had a free apical part that reached 0.483 0.11 um and 0.396 0.09 um.

### Tongue (*Lingua*)

#### Gross and morphometric analysis

**Fig. 7:**
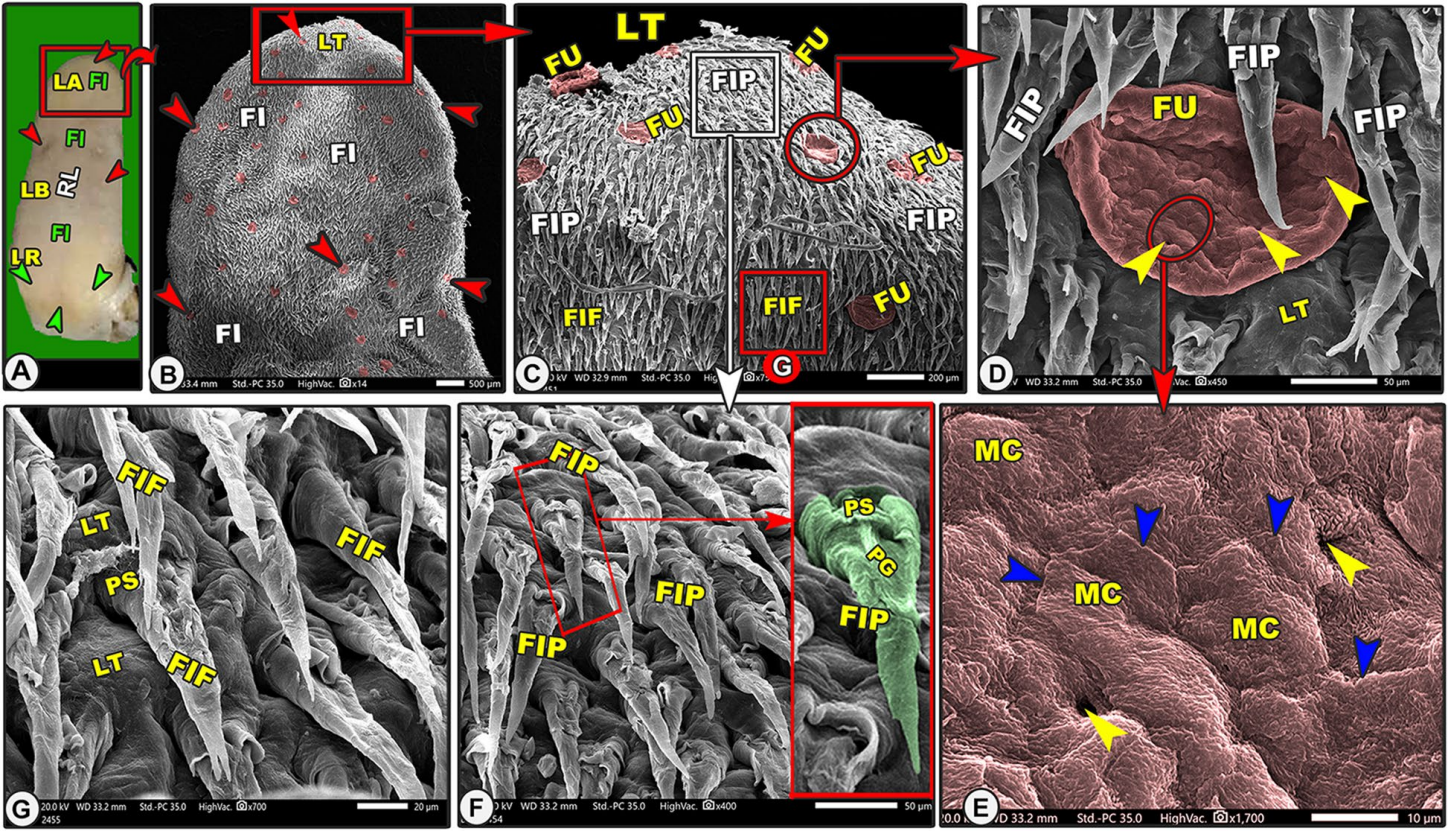
Gross (View **A**) and scanning electron microscopic (Views **B**–**G**) images of the tongue of the Egyptian long-eared hedgehog. The lingual apex (LA) with its filiform (FI) and fungiform (red arrowheads) papillae, body (LB) with its slightly projected median ridge (RL), and root (LR) are clear. Note that the lingual tip (LT) carried round fungiform papillae (FU) with their taste pores (yellow arrowheads) and microplicae cells (MC) with their borders (blue arrowheads). The pointed (FIP) and bifurcated (FIF) filiform papillae with their base (PS), groove (PG), and lingual tubercles

Grossly, the elongated tongue had three main parts; the round apex, the body, and the root (Figs. 7A, 9A, 10A-B/LA, LB, LR). The Egyptian long-eared tongue had a slight elevated lingual ridge on the middle portion of the lingual body (Figs. 7A, 10A/LR). The dorsal epithelium's surface carried numerous lingual papillae. The filiform papillae were the prominent ones, with a high number of the fungiform papillae scattered among them (Fig. 7A/FI, green arrowheads). In addition, there were three ovoid circumvallate papillae on the caudal half of the lingual root in the form of a triangular appearance, of which two were laterally located and one was centrally located towards the pharynx (Figs. 7A, 10A-B/green arrowheads). The ventral surface of the tongue was connected to the sublingual floor by a thick lingual frenulum, leaving a free mobile lingual apex. The lingual papillae were completely absent on the ventral surfaces of both species.

Morphometrically, the lingual thickness gradually increased toward the pharynx, while the lingual width increased gradually toward the body and then decreased again toward the lingual root. The average tongue length was noted to correlate with the feeding lifestyle (0.084 ± 0.012). These findings are in line with the long-eared hedgehog's feeding habits, which call for a greater tongue extension to capture moving or flying insects. The different lingual dimensions were noted in Table 3

**Table 3:** Represent the average dimensions of the tongue (mm) of the Egyptian long-eared hedgehog (*Hemiechinus auratus aegyptius*)

Tongue dimension	Mean ± SD (mm)
The total length of the tongue	**2.3 ± 0.32**
Width of the tongue (at its apex)	0.46 ± 0.02
Width of the tongue (at its body)	**1 ± 0.24**
Width of the tongue (at its root)	0.84 ± 0.17

#### Scanning *electron* microscopic observations

**Fig. 8:**
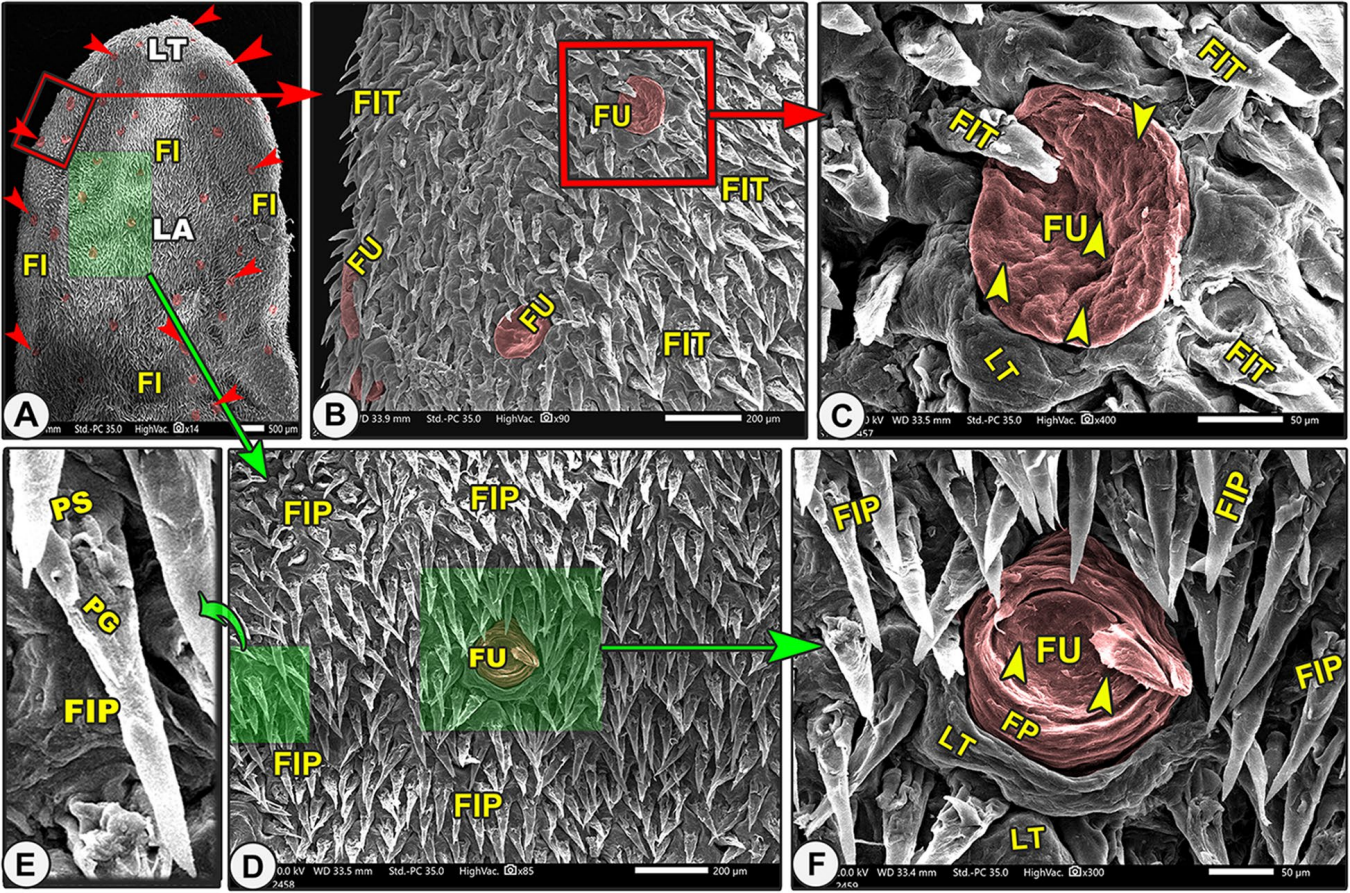
Gross (View **A**) and scanning electron microscopic (Views **B**–**G**) images of the tongue of the Egyptian long-eared hedgehog. The lingual apex (LA) with its tip (LT), filiform (FI), and fungiform (red arrowheads) papillae are clear. The oval fungiform papillae (FU) with their taste pores (yellow arrowheads) and annular pad (FP) are clear. The pointed (FIP) and triangular (FIT) filiform papillae with their base (PS), groove (PG), and lingual tubercles are clear

**Fig. 9:**
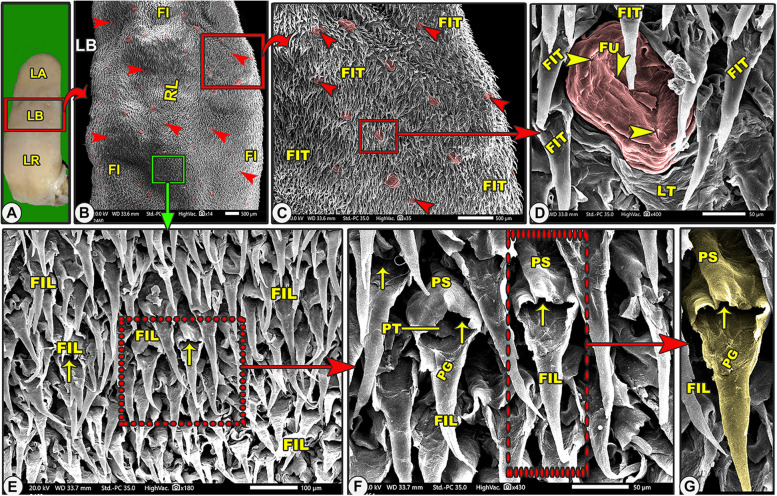
Gross (View **A**) and scanning electron microscopic (Views **B**–**G**) images of the tongue of the Egyptian long-eared hedgehog. The lingual apex (LA), body (LB), slightly projected ridge (RL), filiform (FI), fungiform (red arrowheads) papillae, and root (LR) were clear. Note the fungiform papillae (FU) with their taste pores (yellow arrowheads), while there were triangular (FIT) and leaf-like (FIL) filiform papillae with their base (PS), groove (PG), basket-like base (yellow arrow), papillary tubercle (PT), and lingual tubercles (LT)

**Fig. 10:**
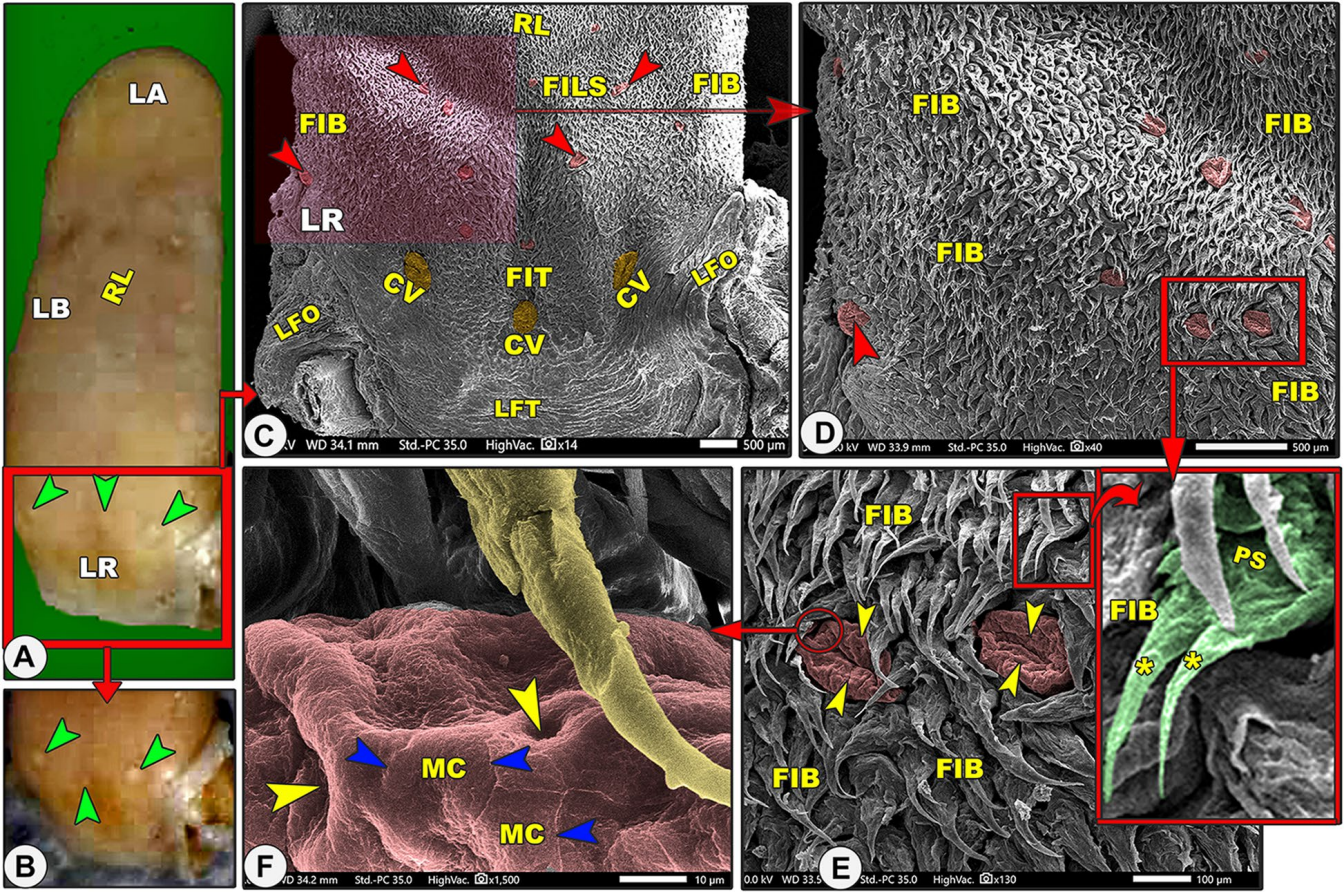
Gross (View **A**) and scanning electron microscopic (Views **B**–**G**) images of the tongue of the Egyptian long-eared hedgehog. The lingual apex (LA), body (LB) with a slightly projected ridge (RL), and root (LR) with their circumvallate papillae (green arrowheads, CV), longitudinal (LFT), and oblique (LFO) lingual folds were clear. Note the fungiform papillae (red arrowheads) with their taste pores (yellow arrowheads), microplicae (MC), and their borders (blue arrowheads). The branched (FIB), triangular (FIT), and small pointed (FILS) filiform papillae with their base (PS) and two processes (stars) were clear

**Fig. 11:**
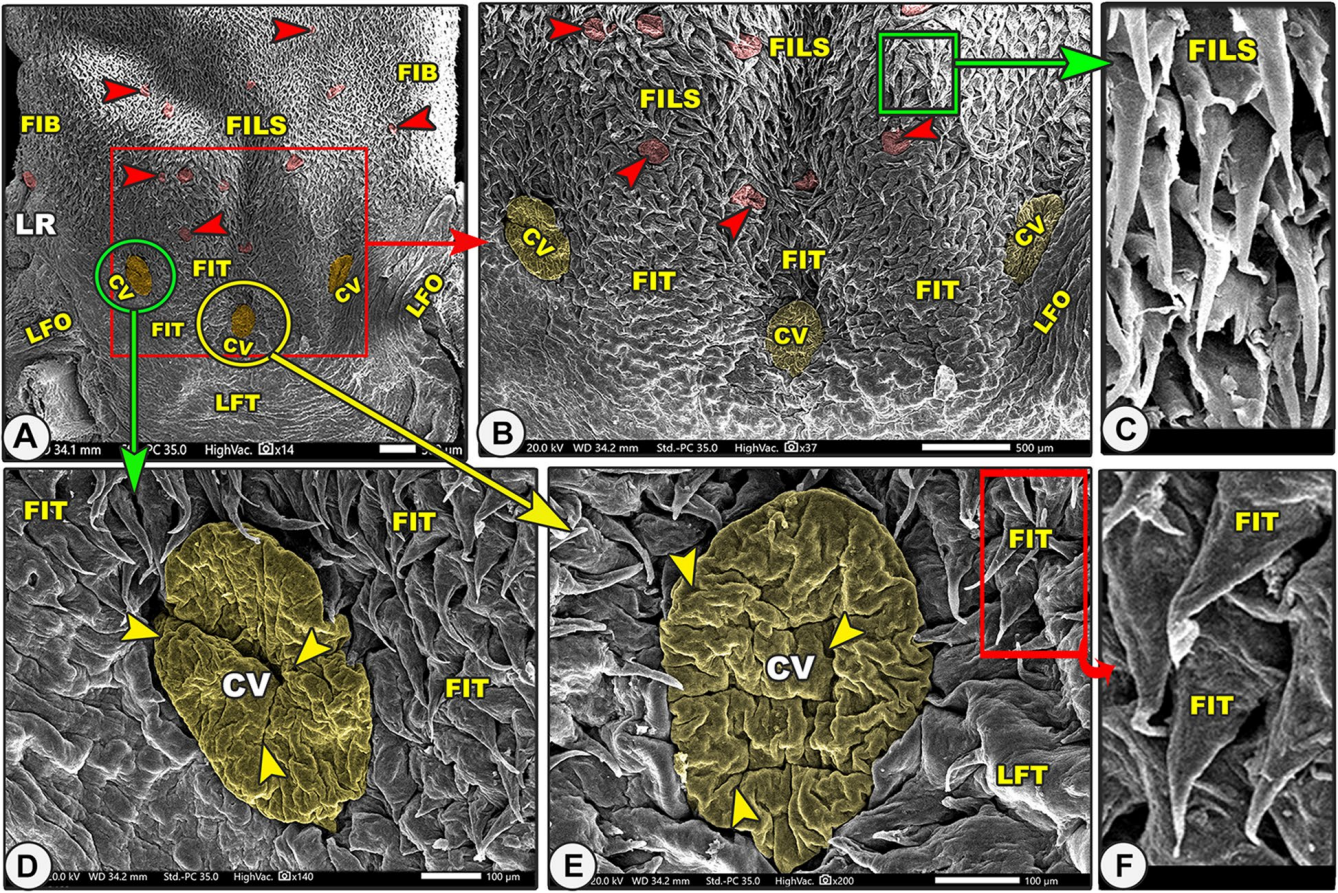
Scanning electron microscopic image of the lingual root of the Egyptian long-eared hedgehog. The lingual root (LR) with its fungiform papillae (red arrowheads), branched (FIB), triangular (FIT), and small pointed (FILS) filiform papillae, circumvallate papillae (CV) with their taste pores (yellow arrowheads), longitudinal (LFT), and oblique (LFO) lingual folds were clear

The dorsal lingual surface had many lingual papillae with various directions, shapes, ultrastructures, functions, and positions, while the ventral lingual surface was smooth and devoid of any lingual papillae. Functionally, on the dorsal lingual surface, two types of papillae were found: mechanical and gustatory papillae, which were found to serve different purposes. On the dorsal lingual surface, one mechanical (filiform) and two gustatory (fungiform and circumvallate) papillary types were identified. These results were macroscopic general information about the tongue, not scanning electron microscopy (Figs. 7B, 8A, 9B, 10C, 11A-B/FI, red and green arrowheads, CV). All the lingual papillae might be oriented in one of three directions: caudal, caudomedian, or median. To help in the collection of food sources in the median region, the lingual papillae, which are found in the lateral lingual region, exhibit caudal, mediocaudal, and/or medially directional orientations. The middle area and lingual tip-located papillae displayed a caudal orientation toward the pharynx and the lingual root.

#### Mechanical filiform lingual papillae

There were six filiform papillary subtypes that were recognized on the tongues of adult Egyptian long-eared hedgehogs that were illustrated as follows:

#### Pointed filiform papillae

These caudally oriented papillae were found on the dorsal surface of the tip, the rostral border of the tongue (Fig. 7B-D,F/FIP), and the median region of the lingual apex (following the bifurcated ones) (Fig. 8D-F/FIP). Each papilla had a wide base, and then its width decreased gradually toward the papillary apex until it was terminated by the pointed tip. The dorsal surface of the papillary body had a slight groove that disappeared at the papillary apex. In addition, there are some micro-ridges (Figs. 7F, 8E/FIP).

#### Triangular filiform papillae

These papillae were found on the lateral lingual region of the apex and body (Figs. 8A-C and 9B-D\FIT) in addition to the circumvallate region (Figs. 10C, 11A-B, and D-F/FIT). The papillae on the apex had a broad end, and those on the body and circumvallate region had a pointed end. The slightly curved dorsal papillary surface had some micro-scales. These papillae had a variety of orientations, with some oriented in the median, others in the caudomedian, and still others in the caudal.

#### Bifurcated filiform papillae

These caudally oriented papillae were found on the median region of the lingual tip only (Fig. 7G\FIF). Each papilla had a wide base with a slightly curved body, forming a median groove, and was terminated by a bifid end (Fig. 7G\PS).

#### Leaf-like filiform papillae

These caudally directed papillae were found on the median region of the body (Fig. 9E-G/FIL)*.* Each papilla had a round base and contained a median tubercle surrounded by a circular edge with a slightly curved dorsal papillary surface forming a groove and terminated by a pointed apex (Fig. 9F-G/FIL, PS, PG, PT, yellow arrow).

#### Branched filiform papillae

These caudally oriented papillae were found on the lateral region of the lingual root (Figs. 10C-E, 11A-B/FIB). Each papilla had a wide base with two papillary bodies of a pointed apex (Fig. 10E/FIB, PS, yellow star).

#### Small pointed filiform papillae

These caudally oriented papillae were found on the median region of the lingual root except the circumvallate region (Figs. 10C, 11A-C/FILS). Each papilla was terminated by a pointed apex.

#### Gustatory lingual papillae

There were two gustatory papillary types (fungiform and circumvallate) that were found on the dorsal lingual surface.

#### Fungiform papillae

On the dorsal surface, there were several fungiform papillae scattered amid the filiform papillae and elevated lingual tubercles (Figs. 7B-E, 8, 9B-D, 10C-F, and 11A-B/Fu). Each dorsal surface of the fungiform papilla had a concave surface and exhibited a microplicae system with unique cell outlines on the irregular surface of these papillae (Figs. 7E, 10F/MC, blue arrowheads). There were two papillary subtypes: the oval and round papillae. The numerous *oval fungiform papillae* were found on the rostral border of the tip, lateral region of the lingual apex, body, and root (Figs. 7C, 8A-B, 9B-C, 10C-D, and 11A/FU). Each microplicae system on the dorsal papillary surface had 4–5 taste pores within the cell outlines (Figs. 7E, 10F/MC, blue and yellow arrowheads). The small numbers of the small round fungiform papillae were found on the median region of the lingual apex and body only (Figs. 8D-F and 9D/FU). Each papilla was surrounded by a circular pad with little microscales and 2–3 taste pores (Figs. 8D-F and 9B/FP, yellow arrowheads).

#### Circumvallate papillae

On the dorsal surface of the caudal part of the lingual root, there were three ovoid circumvallate papillae arranged triangularly. The two laterally situated papillae had a caudomedian direction, while the median centrally situated papilla had a caudal direction (Figs. 10C, 11A-B/CV). These papillae were surrounded by the triangular filiform papillae (Figs. 10C, 11A-B/CV, FIT) and followed caudally by the region of the transverse lingual folds (Figs. 10C, 11A/CV, LFT). The dorsal surface of each papilla was highly irregular and carried numerous micro-folds with five to six taste pores (Fig. 11D-E/red arrowheads). Each circumvallate papilla consisted only of an ovoid bulb without a pad or groove.

#### Gross and SEM morphometric analysis

**Fig. 12:**
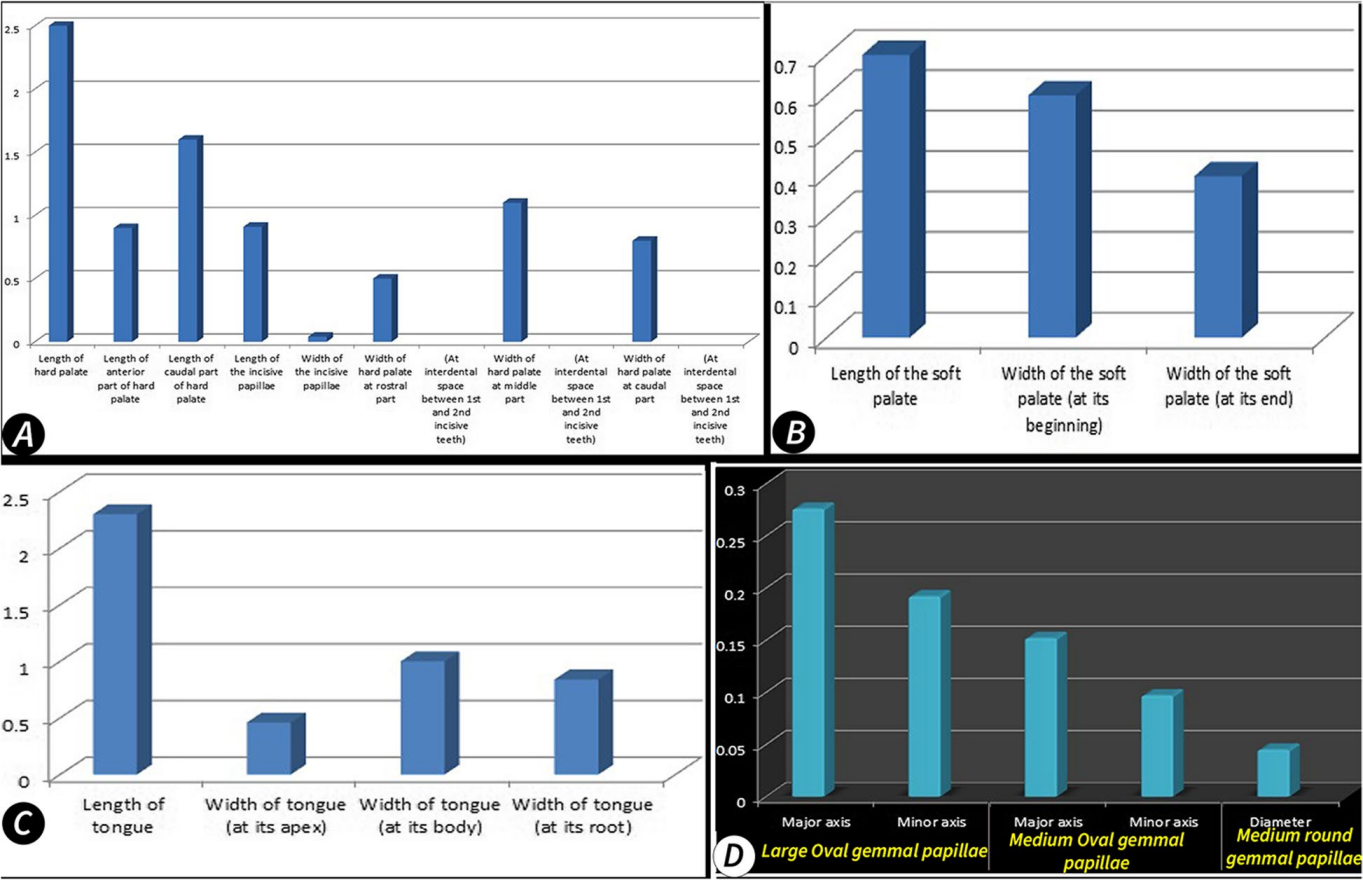
Represent the average gross length and wide of the palate and tongue (Views **A**–**C**) and the SEM morphometric of the Gemmal papillae on the soft palate of the Egyptian long-eared hedgehog. The values were represented as the mean (*n* = 5)

The gross and SEM morphometric analyses of the oral cavity contents were represented in Tables 1, 2, and 3 and Fig. 12.

## Discussion

This study was prepared to describe the oral cavity's gross and ultrastructural features in a *Hemiechinus auratus aegyptius* whose ultrastructure of the lips, sublingual caruncles, hard palate, and soft palate had not been thoroughly described. It also aimed to relate these features to this species' insectivorous feeding habits and make comparisons with other hedgehog species. *Hemiechinus auratus aegyptius*, a recently classified pet animal, had limited knowledge about its lingual morphological adaptations, particularly its lips and palate, compared to typical companion pet animals [4]. Generally, the most published data are focused on the tongue with different methodologies, including gross, histological, and electron microscopic features [2, 11–14, 16], while the morphological adaptations of the lips and the palate are neglected, except in some recently published data [7]. In addition, with the exception of certain older studies, the soft palate is the oropharyngeal cavity's most ignored region in all mammals [27]. Even though *H. auratus aegyptius'* tongue histological characteristics have been thoroughly studied [4, 28], more information is still needed regarding the scanning electronic characteristics of other oral cavity contents, including the tongue, sublingual space, lips, and palate, and to illustrate its adaptations to Egyptian regions. The contents of the oral cavity have various morphological and structural adaptations for various functions like catching, fixing, manipulating, and transporting food particles [10, 29].

Our findings revealed that the elongated upper lip carried a distinctive, previously unreported elongated T-shaped snout-like structure; however, the lower lip showed the slightly protruding cell outlines on an irregular surface under high SEM magnification. The T-shaped snout-like structure is created from a rostrally convex cap-like structure that is joined by two longitudinal columns dividing the upper lip into halves (right and left). Functionally, we suggested that this T-shaped snout-like structure had a main role in the searching (with numerous tactile hairs), collection, and choice of the desirable food particles to be caught by large incisive teeth to suit its own way of life in underground digging and trenches. However, other mammalian species have mobile lips [30]. The palate morphology varies across species due to ecological habits, and it is divided into hard and soft parts [30]. Our study revealed that the hard palate is divided according to the shape and curvature of the palatine ridges into two parts: the triangular, small rostral part and the large quadrilateral caudal part. The same division of the hard palate was described previously [31, 32]. Our high SEM magnification revealed that the caudal palatine part carried a small number of small conical papillae, whereas Tomiate, et al. [33] in Guinea pigs described that the hard palate has conical projection in its rostral part and the lateral sides of the caudal part, but the middle area of the caudal part has numerous filiform papillae.

The current study revealed that the rostral palatine part is named the diastemal palate due to its elongation by the space "diastema" that existed between the incisors and molariform teeth, in addition to the presence of the T-shaped snout-like structure. The diastemal palate has been reported in some mammalian species, including the African giant rat, the Anatolian bobcat, and the red-rumped agouti [30, 34]. Our findings revealed that the triangular rostral palatine part is extended from the lingual surface of the incisor teeth and the caudal end of the longitudinal columns of the snout-like structure rostrally to the rostral border of the second premolar teeth, while the quadrilateral caudal part is extended from the end of the rostral part to the beginning of the rostral border of the 2nd incisive tooth pair and the caudal edge of the last palatine ridge. While in the rabbit, the rostral palatine part is extended from the incisor teeth rostrally to the rostral border of the 1st premolars, and its caudal part is extended from the 1st premolar to the 1st molars [32].

The current study described a characteristic palatine raphe that had not been described previously in other mammalian species, in which it completely disappeared in the rostral palatine part and instead there was a median tubercle, while the caudal part had a median slightly projected raphae in the form of a crest. Moreover, the raphae is in the form of a groove in certain animal species, including herbivorous animals [32, 34]. Palatine ridges are a characteristic structure on the hard palate of most animals [30, 32], but these ridges may be missed in the short hard palate of rocky cavy and Guinea pigs [33, 35]. Our findings revealed that the palatine ridges were absent from the rostral semicircular area of the rostral narrow palatine part, but they were three or four ridges without a raphe in the middle part, and the caudal part had seven or eight ridges. Palatine ridges' adaptations are influenced by various feeding habits, leading to variations in their number [32]. Our findings noted 10–12 pairs of ridges. Morphofunctionally, the herbivorous animals have a large number of ridges, such as rabbits with 14–16 pairs [32] and 17 or 18 pairs in Collared Peccary [36].

The morphological adaptations extended to the palatine ridge shape. Our findings noted smooth palatine ridges. However, the Collared Peccary's hard palate contains transversely oriented palatine rugae connected by a raphe, with the first pair typically being larger than the following pairs [36]. The morphological adaptations extended to the palatine ridges' direction among mammalian species. Our findings noted that the first three or four ridges were strongly convex rostrally, while the following 7 or 8 ridges were nearly straight or slightly curved rostrally with a caudolateral direction, except the last ridge had an anterolateral direction. Meanwhile, Mahdy and Mohammed [32] described the first three pairs in rabbit as having an inverted “V” shape, the fourth pair as having a minor caudal concavity, the fifth pair as being transverse, the sixth through twelfth pairs as diverging rostrally and laterally, and the final three pairs as having a transverse orientation with a straight course. The position of ridges during swallowing aids food particles in moving towards the oropharynx, with more ridges in caudal directions enhancing the hard palate's effectiveness in pushing food backwards [2, 32, 37].

The incisive papilla, which denotes the start of the rugae, is a common feature of the hard palate in all animals [7, 23, 37]. Our findings revealed that this papilla is located at the median position of the rostral palatine part, just parallel to the lingual surface of the 1st pair of incisive teeth, until the beginning of the rostral border of the 2nd pair of incisive teeth. The same incisive papilla position is reported in rabbits [32]. Our findings revealed the flanked papilla on both sides by a groove and an oblique fissure, similar observations in accordance with previously recorded data in rabbits, goats, and foxes [8, 32], while the grooved arc-shaped papilla has four small grooves in a caudorostral direction in rats [38]. Intriguingly, the incisive papilla shape shows some variations among mammalian species, in which our findings revealed the diamond papilla, similar to that described in rabbits [32]. Moreover, rabbits have a small round median elevated papilla [32], and rats have a triangular papilla [38]**.**

The current gross observations revealed that the soft palate is extended from the beginning of the rostral border of the 2nd pair of molar teeth and the caudal edge of the last palatine ridge rostrally to about the middle of the epiglottis caudally. The soft palate extension to the middle of the epiglottis is also reported in rabbits [32]. The uneven, soft palate's oral surface has multiple oblique grooves and folds in the caudomedian direction, which are interconnected by short, small transverse ridges, similar to those described in rats [38]. Our findings noted that the soft palate had numerous palatine gland openings, especially at its caudal part. The presence of the palatine gland openings is described in certain animals [32–34]. Our findings revealed that the soft palate's oral surface carried 12–16 oval or round Gemmal papillae; additionally, their papillary surface carried three to four taste pores with a microplicae system with unique micropores. Among the animals in which the Gemmal papillae of the soft palate are reported are hedgehogs [27] and rabbits [39]. Meanwhile, the soft palates of rats and rabbits are smooth without papillae [32, 38], while they are rough without papillae in Guinea pigs [33]. Our findings reported that the crescentic sublingual space is divided into two lateral and one rostral prefrenular recesses, similar to those reported in numerous animal species [37].

Grossly, our findings revealed that there were three lingual divisions: the apex, body, and root, similar to those reported in most mammals [4, 16, 40–43]. Our morphometric findings revealed that the tongue thickness gradually increased towards the pharynx, while the lingual width increased gradually toward the body, then decreased again towards the root, reaching (0.46 ± 0.02 cm, 1 ± 0.24 cm, and 0.84 ± 0.17 cm at the apex, body, and root, respectively). However, Jackowiak and Godynicki [44] described that the bank vole tongue is wider at the apex (3–4 ml) and root (3 ml) than the body (2 ml), whereas the African giant rat has a wide rostral part and a narrow caudal part [30]. The wedge-shaped tongue is observed in degu, agouti, lesser hedgehog tenrec, and porcupine [13, 14, 43, 45], while the triangular elongated tongue with a narrow middle region is observed in agouti and Zaedyus pichiy [14, 46], but the elongated tongue with a wider apical region is observed in bank vole [47]. The hedgehog's tongue resembles a cylindrical structure that is raised from the sides [16, 42].

The lingual apex shape is correlated with the feeding mechanisms of the different mammals [10, 24, 26]. Our findings revealed the round apex is similar to those described in European hedgehogs, African pygmy hedgehogs, Japanese water shrews, and greater Japanese shrew-moles [16, 42, 48, 49], while the sharp apex is noted in Zaedyus pichiy [46], the shovel-shaped apex in the bank vole [44], and the short pointed apex in the feathertail glider [50]. The current SEM findings described the slight elevated lingual ridge on the middle portion of the body, along with numerous tubercles among the papillae, helping the palatine ridges fix the caught food particles inside the oral cavity, similar to that reported in Egyptian long-eared hedgehogs [4]. Meanwhile, this structure is absent in other hedgehog species, such as the African pygmy hedgehog, the lesser hedgehog tenrec, and the European hedgehog [16, 42, 43]. However, the most herbivorous mammalian species have a lingual prominence at the end of the body [3, 9, 12, 13, 18, 19, 44, 51]. Nonetheless, the median lingual sulcus is absent in the current study, as in Egyptian long-eared hedgehogs, African pygmy hedgehogs, European hedgehogs, and Egyptian fruit bats [4, 16, 40, 42, 51], whereas this median sulcus is observed in the rabbit, rat, agouti, Persian squirrel, agouti, and bank vole [3, 11, 12, 14, 44].

One structural adaptation is the occurrence of different lingual papillary types that serve both mechanical and gustatory roles [10], and the papillary adaptations are extended to include the numbers, shapes, orientations, and dispersion of papillae [2, 51]. The current findings revealed three papillary types: one mechanical (filiform) and two gustatory (fungiform and circumvallate), similar to those described in the Middle East blind mole rat [9]. While the three papillary types are the filiform, fungiform, and foliate papillae in Cape Hyrax [52], the four papillary types (filiform, conical, fungiform, and circumvallate) are observed in African pygmy hedgehog, lesser hedgehog tenrec, and Japanese grass vole [42, 43, 53], whereas the four papillary types are one mechanical filiform and three gustatory (fungiform, foliate, and circumvallate) papillae are observed in Persian squirrel [11]. The five papillary types (filiform, conical, fungiform, circumvallate, and foliate) are recorded in the house mouse, Patagonian cavy, and bank vole [47, 54, 55]. These variations may be related to dietary variances, feeding patterns, and how food is handled in the mouth [40].

Mammalian species exhibit significant variability in their mechanical filiform papillae, which are the most typical lingual structure altered to fit unique feeding methods, exhibiting species- and region-specific traits, and these characteristic features appear in their shape, subtypes, numbers, forms, orientations, and placements [2, 40, 51]. Our findings illustrated six filiform subtypes that were recognized as: pointed, triangular, bifurcated, leaf-like, branched, and small pointed papillae. Our study suggested that the variation in the number of filiform papillae subtypes may be explained by sporadic insectivore behavior in its biotope. According to the current functional hypothesis, the characteristic filiform papillae's six subtypes played important roles in swiftly catching insects during nimble airborne maneuver flights and the movement of food substances and water into the esophagus, as well as the ingestion of food particles [20]. Meanwhile, in the African pygmy hedgehog, there are numerous small conical papillae on the lingual apex while the filiform papillae are on the lingual body only [42], while in the lesser hedgehog tenrec, there are cornice-like filiform papillae on the apex, cornice-like filiform papillae with two processes on the body, and crown-like filiform papillae with 2–5 processes on the rostral part of the root [43].

The filiform papillae display a variety of subtypes in accordance with how well they are adapted to particular feeding mechanisms under various environmental conditions [2]. There are different numbers of filiform subtypes in various mammals; for example, our study revealed six filiform subtypes, similar to those described in Egyptian fruit bats [1]. The seven subtypes are observed in some bats [56], the five subtypes in the Egyptian mouse‐tailed bat [2], the four subtypes in Egyptian fruit bats and Japanese grass vole [40, 53], three subtypes in Egyptian long-eared hedgehogs, lesser hedgehog tenrec, and nutria [4, 43, 57], the two subtypes in the bank vole and Brandt's hedgehog [47, 58], but only one papillary subtype was observed in the African pygmy hedgehog and Persian squirrel [11, 42]. Typically, filiform papillae are seen to cover the dorsal surface of insectivores' tongues, even reaching the central part of the structure [16].

On the other hand, the shapes and subtypes of the filiform system may be influenced by the animal's geographic dispersion in relation to the food particles and environmental circumstances that are available [20]. Comparing hedgehogs from different regions of the same country or other countries with those from the current study made this anatomical reality evident. The current findings revealed six subtypes of the lingual filiform of the Egyptian long-eared hedgehogs collected from Egypt's Matrouh Governorate, while the hedgehogs collected from the Abu-Rawash region of Egypt's Giza Governorate carried three subtypes of the filiform papillae [4]. Meanwhile, the hedgehogs collected from Iran's Kermanshah city have two subtypes of filiform papillae [58], whereas African pygmy hedgehogs [42] collected from Slovenia have only one filiform papillary type on the body and a small conical papillae on the apex. This anatomical fact is clear in the Egyptian fruit bats, in which those collected from Egypt carry six filiform subtypes [1, 20], while those collected from Japan carry five subtypes, and those collected from Saudi Arabia carry four subtypes [40], whereas those collected from Poland carry three subtypes [59]. The current findings supported earlier research suggesting that the architectural characteristics, geographic distribution, and number of the lingual papillae are distinct and represent each mammalian clade's evolutionary taxonomic rank [40].

The numbers, distributions, shapes, and dorsal surface ultrastructure of the fungiform papillae differ between mammals. Generally, in all mammals, the fungiform papillae are scattered among the filiform papillae and elevated lingual tubercles [2, 16, 40, 49]. In higher animal orders, the tongue has a significant number of fungiform papillae uniformly distributed between filiform papillae throughout the entire apex and body [17–19]. Generally, the most papillary accumulation is observed at the apex in most mammals, including the studied animals that have a major role in food particle selection [2]. Functionally, the lingual apex is referred to as a unique sensory organ for tasting food substances to decide whether or not to accept them [51]. Our findings revealed two papillary shapes: the oval and the round. Meanwhile, most mammalian tongues have only one shape, but they vary in shape in some animals; the dome-shaped papillae are observed in the European hedgehog, African pygmy hedgehog, Cape hyrax, Persian squirrel, Zaedyus pichiy*,* Greater Japanese Shrew-mole, agouti, and Egyptian long-eared hedgehog [4, 11, 14, 16, 42, 46, 49, 52]. The quadrilateral papillae are found in the Egyptian fruit bat [40], the round papillae in bank vole and Egyptian mouse‐tailed bat [2, 47], elliptical or circular papillae in bats [60], discoidal papillae in Brandt's hedgehog and Egyptian tomb bat [40, 58], volcano-like papillae in agouti [14], and the rectangular and round papillary shape are reported in the Egyptian fruit bat [1].

Our findings revealed two fungiform subtypes: the numerous oval ones on the rostral border of the tip, lateral region of the apex, body, and root, and the little, small round ones on the median region of the apex and body only. Meanwhile, the fungiform papillae are found on the lingual body and root but become small at the apex and are usually seen on the apex margins of European hedgehogs [16]. Fungiform papillae are present throughout the whole dorsal lingual surface of bats [60]. Compared to the common shrew, which only has them on the body [61], the Egyptian mouse‐tailed bat and Egyptian fruit bat have fungiform papillae on the apex and lateral edges [2, 51]. However, fungiform papillae are thinly dispersed in the center and/or at the apex's borders, and they become less in number nearer the root in the Japanese shrew-mole and large Japanese mole [49, 62]. Unlike the Japanese shrew-mole and furry-snouted mole, which have them in the middle or at the edges [49]. The house musk shrew and the large Japanese mole, Egyptian mouse‐tailed bat, and Egyptian fruit bat do not have them in the middle of their tongues [2, 16, 51]. On the apex, the furry-snouted shrew mole is missing them [62], while the Shinto shrew and long-clawed shrew lack them on the lingual apex, but the tongue center, the apex, and the caudal lingual region of the Japanese water shrew and Dsinezumi shrew are devoid of fungiform papillae [48]. Moreover, these papillae are located at the center and lingual tip of the bank vole and Japanese grass vole [63, 64]. On each half of the tongue, four rows of fungiform papillae are grown on the dorsal and dorsolateral surfaces in lesser hedgehog tenrec [43].

Our SEM magnification findings noted that the dorsal surface of fungiform papillae had a concave surface and exhibited a microplicae system on the irregular surface, with 4–5 taste pores on the oval ones and 2–3 taste pores on the round ones. While the smooth papillary surface with numerous taste pores is observed in Cape hyrax [52], the high magnification of the concave papillary surface reveals taste pores between the shingle-like flattened cells of a stratified squamous epithelium in the Brandt's hedgehog [58]. While the fungiform papillae missed the taste pores on the tongue of porcupines [15, 65]. There is no connection between the arrangement and number of circumvallate papillae, on the one hand, and the environment and feeding practices, on the other. The number of circumvallate papillae varies among mammal taxa [10]. Both the Cape Hyrax and the blood-drinking bats are completely devoid of them [52, 66]. In the rat, mouse, Japanese grass vole, and bank vole, just one is found on the midline of the lingual root [4, 44, 53]. Two papillae are observed in the majority of animals, such as the rabbit, Middle East blind mole rat, and degu [3, 9, 13]. Three circumvallate papillae have been observed in our study and in the Egyptian fruit bats, American beaver, Persian squirrel, African pygmy hedgehog, lesser hedgehog tenrec, and European hedgehogs [1, 11, 16, 42, 43, 67], but the four papillae were observed in the agouti [14].

Our findings found that the caudal part of the lingual root had three ovoid circumvallate papillae, arranged triangularly: two lateral papillae and the median papilla. The circumvallate papillae's triangular arrangement is limited to observations in some mammals such as Egyptian long-eared hedgehog, Egyptian fruit bat, Egyptian mouse‐tailed bat, Egyptian tomb bat, lesser hedgehog tenrec, feathertail glider, and Persian squirrel [1, 2, 4, 11, 40, 43, 50, 51], while these three papillae take the V-appearance in African pygmy hedgehog [42]. There are some minor variations in the papillary shape in which they are elliptical in some mammals such as Japanese water shrew [48], egg-shaped papilla in the long-eared hedgehog [28], round in the feathertail glider [50], whereas they are oval in the house musk shrew, Japanese mole [62], bank vole [47], *Zaedyus pichiy* [46], degu [13], and European hedgehog [16], but the four papillae appeared oval to elongated in agouti [14] and in greater Japanese shrew‐mole [49].

The current study showed that the circumvallate papillae were represented only by their bulbs, without any annular groove or pad. Meanwhile, in ruminant species and the Persian squirrel, these circumvallate papillae have a papillary bulb that is surrounded by both a groove and a pad [18, 19, 68]. The papillae are surrounded only by a groove named the circumpapillary groove in the following animals: Brandt's hedgehog, lesser hedgehog tenrec, African pygmy hedgehog, Middle East blind mole, American beaver, and porcupine [9, 42, 43, 45, 58, 67], while the degu, gouti, and Patagonian cavy are intermittent, which led to the formation of two grooves [14, 53, 55]. The fungiform papillae in the European hedgehog and Brandt's hedgehog papillae are encircled by a circular flat pad and a continuous ridge that divides the papillary body from the tongue surface [16, 58], while these papillae are surrounded by two separate ridges in the Shinto shrew and long-clawed shrew [48]. Our findings showed that the irregular dorsal surface of the circumvallate papilla was highly irregular and carries numerous micro-folds with five to six taste pores. The irregular dorsal papillary surface observed was also described in Brandt's hedgehog, long-eared hedgehog*,* and bank vole [28, 47, 58], whereas the smooth dorsal papillary surface is noted in the fox [69].

## Conclusion

The current study focused on the anatomical characteristics of the Egyptian long-eared hedgehog's oral cavity. The upper lip had an elongated T-shaped snout-like structure. The hard palate had a triangular rostral and a quadrilateral caudal part with slightly oblique seven or eight ridges with the palatine raphae. The diamond-incisive papilla was flanked on both sides by a groove. The uneven, soft palate's surface had 12–16 Gemmal papillae. The Gemmal papillary surface had three to four taste pores with gland openings. The dorsal lingual surface had six filiform subtypes; pointed, bifurcated, leaf-like, branched, and small pointed papillae. There are two fungiform subtypes: numerous ovals and numerous rounds. The caudal root part had three circumvallate papillae arranged triangularly.

## Data Availability

The datasets used and/or analyzed during the current study are available from the corresponding author on reasonable request.
